# Cu isotopic signature in blood serum of liver transplant patients: a follow-up study

**DOI:** 10.1038/srep30683

**Published:** 2016-07-29

**Authors:** Sara Lauwens, Marta Costas-Rodríguez, Hans Van Vlierberghe, Frank Vanhaecke

**Affiliations:** 1 Department of Analytical Chemistry, Ghent University, Krijgslaan 281-S12, BE-9000 Ghent, Belgium; 2 Department of Gastroenterology and Hepatology, Ghent University Hospital, De Pintelaan 185-1K12IE, BE-9000 Ghent, Belgium

## Abstract

End-stage liver disease (ESLD) is life-threatening and liver transplantation (LTx) is the definitive treatment with good outcomes. Given the essential role of hepatocytes in Cu homeostasis, the potential of the serum Cu isotopic composition for monitoring a patient’s condition post-LTx was evaluated. For this purpose, high-precision Cu isotopic analysis of blood serum of ESLD patients pre- and post-LTx was accomplished *via* multi-collector ICP-mass spectrometry (MC-ICP-MS). The Cu isotopic composition of the ESLD patients was fractionated in favour of the lighter isotope (by about −0.50‰). Post-LTx, a generalized normalization of the Cu isotopic composition was observed for the patients with normal liver function, while it remained light when this condition was not reached. A strong decrease in the δ^65^Cu value a longer term post-LTx seems to indicate the recurrence of liver failure or cancer. The observed trend in favour of the heavier Cu isotopic composition post-LTx seems to be related with the restored biosynthetic capacity of the liver, the restored hepatic metabolism and/or the restored biliary secretion pathways. Thus, Cu isotopic analysis could be a valuable tool for the follow-up of liver transplant patients and for establishing the potential recurrence of liver failure.

Copper is an essential trace element required as cofactor in many enzymes. However, when it is present in excess, the high oxidative potential of free Cu ions can generate reactive free radicals with subsequent cellular damage. Thus, Cu homeostasis needs to be regulated strongly to maintain cellular Cu concentrations at levels at which both toxicity and deficiency are avoided^1^,^2^,^3^.

The liver, as the principal storage site for Cu, plays a key role in Cu homeostasis. Dietary copper is absorbed in the intestine and transported to the liver *via* the portal blood, where it is taken up by hepatocytes. The copper taken up by the hepatocytes is incorporated into the copper-dependent ferroxidase ceruloplasmin (Cp) and subsequently secreted in the blood for distribution throughout the body. Total body Cu levels are controlled by excretion of excess Cu *via* the bile^1^,^4^,^5^.

Cu status in the body is altered in liver disease, especially when there are obstructions in bile flow and/or impaired protein synthesis^6^,^7^. Studies with radioactive Cu have shown that Cu turnover in the liver increases from 20–30 days in healthy individuals to 600–700 days for patients with biliary cirrhosis^8^. Hepatic Cu overload has been observed in liver diseases that arise from etiologies such as Wilson’s disease (WD)^9^, viral hepatitis^10^, cholestasis^9^ and excessive alcohol consumption^11^. WD is a genetic disorder that leads to Cu accumulation, first in the liver, but ultimately also in the brain and other tissues^12^, and to low or normal serum Cu concentrations compared to controls. In contrast, high or normal Cu concentrations in blood serum can be observed in cirrhosis^13^ and hepatocellular carcinoma (HCC) patients. End-stage liver disease (ESLD) can arise from these etiologies and can be complicated by other life-threatening complications (*e.g.,* hepatic encephalopathy, ascites, portal hypertension and hepatorenal syndrome)^14^,^15^. An elevated risk of HCC and extra-hepatic cancers has been established in patients with cirrhosis^16^.

As a result of the above, Cu levels in serum of ESLD patients frequently overlap with those of the reference population and there is no clear relation between the Cu concentration and the clinical course of the disease. Recently however, the potential of the Cu isotopic composition in blood serum as a diagnostic tool for diseases affecting Cu metabolism has been suggested^17^. Patients with Wilson’s disease^18^, ESLD^19^ and HCC^20^ showed a lighter serum Cu isotopic composition than did the reference population.

Due to the complexity of the liver, there is no single clinical test for assessing hepatic disorders^21^,^22^. A series of tests, for the determination of what is frequently termed “liver function parameters”, is performed for the management of liver diseases. These parameters are used for i) detection of hepatic injury, *e.g.,* aspartate aminotransferase (AST) and alanine aminotransferase (ALT), ii) assessment of hepatic biosynthetic capacity, *e.g.,* albumin (Alb), prothrombin time (PT) and international normalized ratio (INR), and iii) assessment of hepatic metabolism, *e.g.,* bilirubin (Bili)^22^. Liver transplantation (LTx) is the definitive treatment with successful outcomes for patients with ESLD, for patients with HCC development and for patients with acute liver failure (ALF)^23^. In this context, the time on the liver transplant waiting list, the transplant itself and the recovery period are crucial factors determining the outcome of LTx.

The aim of this research was to investigate the Cu isotopic signature in blood serum of patients with ESLD pre-LTx and its evolution post-LTx to evaluate the capability of this parameter for following up the patients and for establishing the potential recurrence of liver failure. For this, high-precision Cu isotopic analysis of ~100 blood serum samples from 32 liver transplant patients was carried out *via* multi-collector ICP-mass spectrometry (MC-ICP-MS), after digestion of the serum and chromatographic isolation of the target element. For each patient, blood serum samples collected pre- and post-LTx were analyzed. In addition, serum samples collected at several time points post-LTx (up to >1 year post-LTx for some patients) were included.

## Results

The study group consisted of 32 patients with ESLD, who underwent LTx between September 2011 and April 2015. The patients were subjected to LTx because of different diseases, *e.g.,* cirrhosis, steatosis, cholestasis, hepatitis, hypertension, amyloidosis, polycystic liver and HCC (Table 1). Information on the patient´s diagnosis, liver function parameters pre-LTx and post-LTx (including bilirubin, AST, ALT, PT, INR and Alb), post-LTx complications and other chronic diseases, is given in the Supplementary Table S1.

The Cu isotopic composition, expressed as δ^65^Cu (±2 s.d.) value, and the Cu concentration in blood serum of the ESLD patients pre- and post-LTx are provided in the Supplementary Table S2. The δ-notation indicates the relative difference (in ‰) between the Cu isotope ratio (^65^Cu/^63^Cu) in the sample and that of the NIST SRM 976 Cu isotopic reference material.

**Figure 1:**
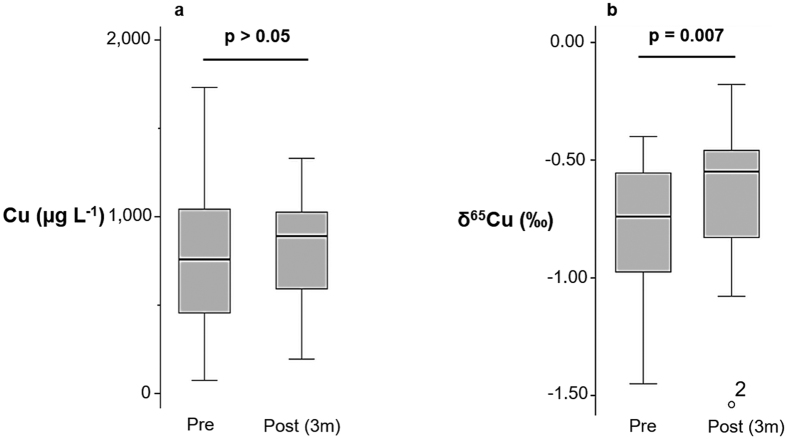
Boxplots of serum Cu concentrations and isotopic composition of liver transplant patients pre-LTx and 3 months post-LTx. Empty circles are outliers. **(a)** Serum copper concentrations (μg L^−1^). No significant change in serum Cu levels post-LTx (p > 0.05). **(b)** Copper isotopic compositions in serum. A significant enrichment in ^65^Cu was observed post-LTx (p = 0.007).

Patients with ESLD (N = 32) showed an average serum Cu level of 750 ± 770 (2 s.d.) μg L^−1^ pre-LTx and 810 ± 580 (2 s.d.) μg L^−1^ three months post-LTx. No significant differences were observed in Cu serum concentrations between the two cohorts (related samples Wilcoxon signed rank test, p > 0.05) (Fig. 1a). However, the Cu isotopic composition in serum of these ESLD patients pre-LTx was fractionated in favour of the light Cu isotope. The same trend was observed in patients who developed HCC^20^, but no significant difference was observed in δ^65^Cu value between the ESLD cohort pre-LTx with HCC (N = 10) and that without HCC (N = 22) (related samples Wilcoxon signed rank test, p > 0.05). The average δ^65^Cu value was −0.80 ± 0.57 (2 s.d.)‰ (N = 32), while the reference range reported for assumed healthy individuals is −0.26 ± 0.40 (2 s.d.)‰ (N = 47)^24^. These results were in agreement with the average δ^65^Cu value reported in our previous work on liver cirrhosis patients (−0.78 ± 0.72 (2 s.d.)‰, N = 25)^19^. Three months post-LTx, the average δ^65^Cu value had significantly (related samples Wilcoxon signed rank test, p = 0.007) risen to −0.63 ± 0.55 (2 s.d.)‰ (N = 32) (Fig. 1b).

**Figure 2:**
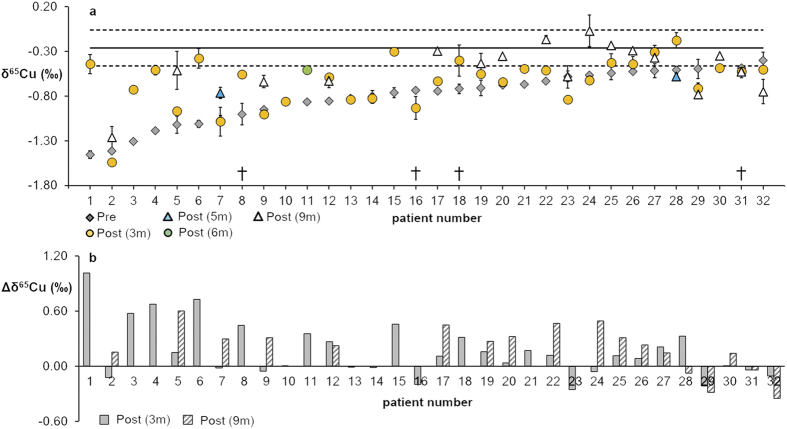
δ^65^Cu and Δδ^65^Cu values in serum samples of pre- and post-LTx (3 and 9 months) patients. **(a)** δ^65^Cu values. Error bars represent 2 s.d. The full line represents the average value for healthy individuals as reported by Albarède *et al*.^24^. The dotted lines represent ± 1 s.d. on this average. For patient 11, a serum sample 3 months post-LTX was not available and a sample collected 6 months post-LTx was included instead. For patients 7 and 28, serum samples 9 months post-LTx were not available and samples collected 5 months post-LTx were analysed instead. The low δ^65^Cu values observed in the ESLD population tend to increase post-LTx. **(b)** Δδ^65^Cu values.

In Fig. 2a, the serum δ^65^Cu values obtained for each patient pre-LTx, 3 months post-LTx and – for some patients – 9 months post-LTx are shown. As can be seen, the serum Cu isotopic composition tends to higher values for most of the patients, *i.e.* the δ^65^Cu value normalizes post-LTx when the patient recovers normal liver condition, in concordance with the recovery of the “liver function parameters” (Table 2 and Supplementary Table S1). About 40% of the patient cohort studied (N = 32) reached the reference range 3 months post-LTx, increasing to ∼60% 9 months post-LTx. In Fig. 2b, the serum Δδ^65^Cu (‰) values, defined as Δδ^65^Cu = δ^65^Cu_post-LTx_ − δ^65^Cu_pre-LTx_, obtained 3 months and – for some patients – 9 months post-LTx are shown. A mean and maximum Δδ^65^Cu (‰) value of respectively 0.16 and 1.01 was observed 3 months post-LTx.

**Figure 3:**
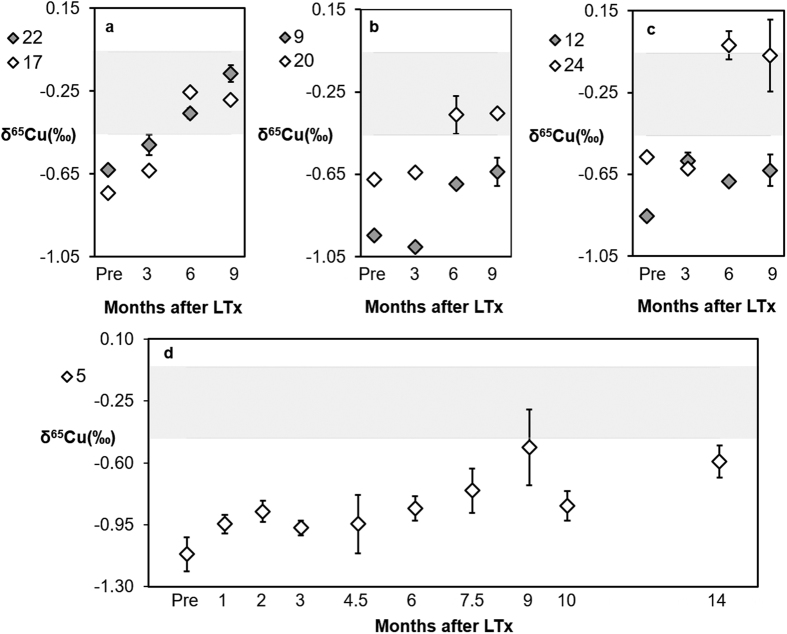
Follow-up of serum δ^65^Cu values in patients without recurrence of liver failure. Error bars represent 2 s.d. The grey area represents the reference range (average value ± 1 s.d.) for healthy individuals^24^. Patients follow a gradual increase in δ^65^Cu post-LTx. **(a)** Patients 17 and 22 **(b)** Patients 9 and 20. **(c)** Patients 12 and 24. **(d)** Patient 5.

**Figure 4:**
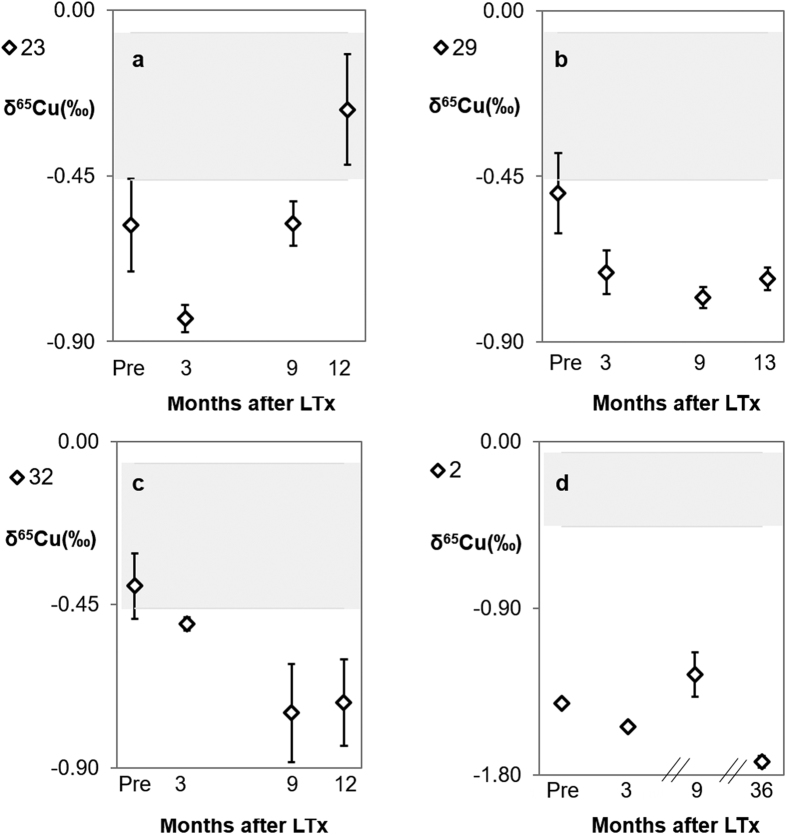
Particular trends observed in serum δ^65^Cu values post-LTx. Error bars represent 2 s.d. The grey area represents the reference range (average value ± 1 s.d.) for healthy individuals^24^. **(a)** Patient 23. **(b)** Patient 29. **(c)** Patient 32. **(d)** Patient 2.

To assess the variations in the Cu isotopic composition further in time post-LTx, the follow-up was extended for twelve patients to ≥9 months post-LTx (Figs 3 and 4). Patients 5, 9, 12, 17, 20, 22, 24 (Fig. 3) and 23 (Fig. 4) showed a gradual enrichment in δ^65^Cu as a function of time, whether or not accompanied with some fluctuations.

Patient 23 (Fig. 4a), diagnosed with polycystic liver disease, showed a normal pre-transplant liver function but post-LTx, a temporary dysfunction of the new liver was established. This is likely the reason for the slight decline of δ^65^Cu ± 2 s.d. (‰) from −0.58 ± 0.13 (pre-LTx) to −0.84 ± 0.04 (3 months post-LTx). After 3 months, δ^65^Cu increases towards the range of healthy individuals with a δ^65^Cu value of −0.27 ± 0.15 (2 s.d.)‰. The same behaviour was observed for patient 32 (Fig. 4c), suffering from familial amyloidosis, a disease characterized by extracellular deposition of amyloid in various organs, leading to multisystem organ dysfunction^25^. This patient showed a normal pre-LTx liver function, but post-LTx, some abnormalities were evident from the liver function parameters. This possibly explains the dropdown in δ^65^Cu ± 2 s.d. (‰) from −0.40 ± 0.09 (pre-LTx) to −0.75 ± 0.14 (9 months post-LTx).

For a few patients (2, 29 and 32), the Cu isotopic composition remained very light, even more than one year post-LTx (Fig. 4). Patient 2, who underwent LTx because of alcoholic cirrhosis and HCC, was an outlier (Fig. 1), showing a serum δ^65^Cu ± 2 s.d. value 9 months post-LTx of −1.26 ± 0.12‰. This patient was monitored up to 3 years post-LTx (Fig. 4d) and at this date, the δ^65^Cu value had further dropped to −1.73 ± 0.03 (2 s.d.)‰. On the basis of the clinical information, this patient seemed to have recovered well from the LTx without complications, recurrent liver disease, and other chronic or genetic disorders. Until now, no clear explanation can be given for these very negative δ^65^Cu values.

Patient 29 (Fig. 4b) underwent transplantation due to hepatitis C virus cirrhosis (HCV C) and was re-transplanted 14 days after the first LTx due to primary non-function. Although the patient recovered completely afterwards, he showed a very prolonged post-operative status with slow recuperation of liver function (Supplementary Table S1). This possibly contributed to the decline in the δ^65^Cu ± 2 s.d. value from −0.50 ± 0.11‰ (before the first LTx) to −0.78 ± 0.03‰ (9 months after the first LTx). In this case, δ^65^Cu value could potentially also have reached the range of healthy individuals at a later time, but unfortunately, no such serum samples were available.

**Figure 5:**
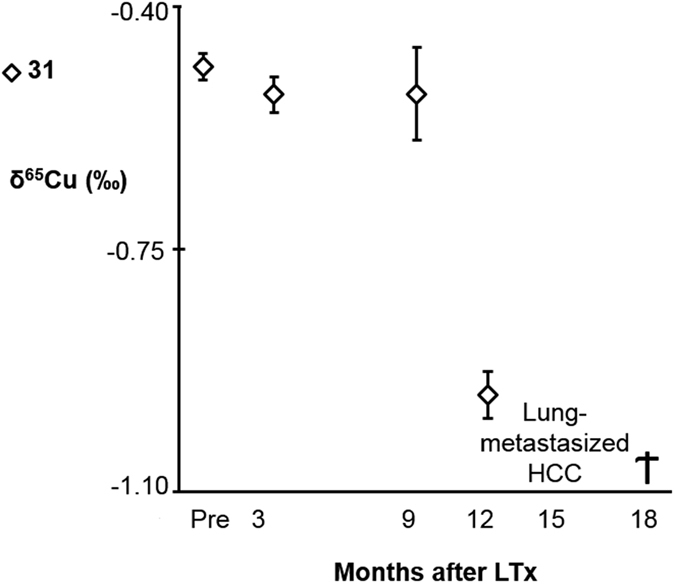
Trend in serum δ^65^Cu value in a deceased patient with HCC. Error bars represent 2 s.d. A tremendous decrease in δ^65^Cu can be observed at 12 months post-LTx. The patient was diagnosed with recurrence of lung-metastasized HCC 15 months post-LTx and deceased 3 months later.

Four patients passed away during the course of this work (8, 16, 18 and 31) and they all showed abnormal trends and/or light Cu isotopic composition, probably related with additional complications and diseases post-LTx. Patients 8 and 16 died at 3 months post-LTx. Patient 8 presented acute renal failure, but the transplanted liver was functioning properly, which is likely the reason of the change in serum δ^65^Cu towards the reference value. Patient 16 deceased due to sepsis and hepatorenal syndrome, which might explain the slight decrease in serum δ^65^Cu following LTx. Patient 18 was diagnosed with recurrence of liver failure 10 months post-LTX and died 23 months post-LTx. However, 3 months post-LTx, no liver disorders were established and the serum δ^65^Cu values had increased within the first months post-LTx. For patient 31 (Fig. 5), who was diagnosed with HCV C and HCC, the δ^65^Cu ± 2 s.d. value pre-LTx (−0.49 ± 0.02‰), approximated the reference range and remained more or less stable within 9 months post-LTx. However, at 12 months post-LTx, the δ^65^Cu value declined very strongly to −0.96 ± 0.03 (2 s.d.)‰ and at 15 months post-LTx, the patient was diagnosed with recurrent lung-metastasized HCC. Since this tremendous change in δ^65^Cu was observed 3 months before the diagnosis of lung-metastasized HCC, this case seems to illustrate the added value of the Cu isotopic composition in blood serum as an additional parameter to assess presence/recurrence of liver disease.

## Discussion

Patients with ESLD showed a significantly lighter serum Cu isotopic composition (by about −0.50‰) than the reference population, in concordance with the fractionation observed previously for another liver cirrhosis patient cohort studied (also by about −0.50‰)^19^. This enrichment in the light Cu isotope was higher in serum from ESLD patients than that reported for HCC patients^20^. However, the wide spread in the δ^65^Cu values in both ESLD and HCC could reflect the heterogeneity of the patient´s condition, *e.g.,* severity of the disease. Even more, some patients had developed HCC and liver biopsies of HCC patients revealed that tumors are systematically ^65^Cu-enriched relative to normal adjacent tissue (by about −0.50‰)^20^, which can enhance the ^65^Cu depletion observed in serum.

Natural variations in the isotopic composition of transition metals in organisms are attributed to differences in coordination environments and in oxidation state. Heavier isotopes favour hard bonds, *i.e.* bonds to amino acids with harder ligands (binding to N or O), while lighter isotopes form preferentially bonds to softer ligands (binding to S)^24^. In serum, Cu is mostly bound to Cp and to a lesser extent to transcuprein, Alb and some low molecular weight components^26^. Since the liver is the major source of most serum proteins, liver disease affects Cp and Alb synthesis, function and redox change, and therefore Cu-binding^27^,^28^. Patients with cirrhosis typically have lower Alb and higher Cp levels^22^. In the liver, Cu and Zn are bound with strong affinity to metallothionein (MT), a cysteine-rich protein which converts these elements into a non-toxic form and acts as chaperone for providing Cu and Zn to several metalloenzymes^29^. Increased hepatic and plasma MT levels have been reported in liver disease associated with increased liver Cu concentration, such as cholestasis. Obstruction of bile flow results in the re-absorption of the cuproproteins from the bile and retention of Cu in the hepatocytes^6^,^7^. Elevated MT concentrations may be related to detoxification of hepatic accumulated copper, which can possibly explain the normal plasma MT concentrations found in liver disease, not accompanied by an elevated hepatic Cu concentration^30^,^31^. Alterations in these proteins can induce the Cu isotope fractionation observed in serum. It has been suggested that low δ^65^Cu values in serum of HCC patients are related with the release of intracellular Cu from cysteine clusters in MT^20^.

The potential of the serum Cu isotopic composition for diagnosis, management of liver disease and prioritization of liver transplants was previously pointed out^19^. Monitoring of ESLD patients post-LTx revealed an increase in the serum δ^65^Cu value, tending to the reference value, *i.e.* normalization, when the patient recovers normal liver function. The fluctuations or slight decreases in the δ^65^Cu values observed in a minority of this cohort, especially at 3 months post-LTx, can be related with abnormal liver function or slow recuperation. A strong dropdown in δ^65^Cu values at long-term post-LTx (*e.g.,* 9 months) can be indicative of a life-threatening condition (Fig. 5). Thus, the serum Cu isotopic composition seems to predict the recurrence of HCC and/or liver failure.

In general, WD patients improve Cu metabolism^32^ and patients with cholestasis have progressive increase in biliary Cu secretion rates and lower levels of urinary Cu after LTx. A disturbance in Cu metabolism and biliary secretion has been observed in the immediate postoperative period, which was restored during the next weeks^33^. A series of surgeries on biliary artresia patients have shown a decrease in hepatic Cu content with the improvement of cholestasis after the establishment of bile excretion^34^. Plasma Alb increased markedly post-LTx and normal or near-to-normal liver function parameters, such as PT, serum bilirubin, AST and ALT, were observed in liver-transplant patients^35^. Even more, it has been shown that products of hepatic synthesis permanently retain the metabolic specificity of the donor^36^. Thus, a general normalization in the Cu isotopic composition following LTx can be the evidence of the restored hepatic biosynthetic capacity, *e.g.,* a normalization in Cp and Alb synthesis, to a restored hepatic metabolism and/or to restored biliary secretion pathways.

In our previous work on liver cirrhosis patients, it was already suggested that δ^65^Cu in serum could be used for the diagnosis and prognosis of liver cirrhosis^19^. The δ^65^Cu values were significantly correlated with bilirubin, AST, PT, INR and Alb. Even more, a significant correlation between δ^65^Cu and the model for end-stage liver disease (MELD) score was found. This exploratory study suggests that Cu isotopic analysis in blood serum could also be a valuable non-invasive tool for the long-term monitoring of a patient’s liver function after LTx and for assessing recurrence of liver disease and HCC.

## Methods

### Samples

Serum samples of 32 patients (28 male and 4 female; age range, 28–70 years) who underwent LTx, were acquired from the Ghent University Hospital (UZGent, Belgium). Blood serum samples were collected within the week or month (two patients) pre-LTx and approximately 3 months post-LTx to evaluate the Cu isotopic signature. For a longer-term follow-up, samples taken up to ≥1 year post-LTx were included for some patients. In total, about 100 blood serum samples were analyzed.

Each blood sample was originally collected in a BC Vacutainer blood tube, suitable for trace element analysis and subsequently centrifuged. After centrifugation, the serum samples were analysed at the Ghent University Hospital for determination of various clinical parameters. An aliquot of about 500 μL of serum sample was transferred to a pre-cleaned Eppendorf tube and stored at −20 °C until sample preparation commenced.

### Sample Preparation

An aliquot of about 500 μL of blood serum was digested using 2 mL of 14 M sub-boiled *pro-analysis* HNO_3_ and 0.5 mL of 9.8 M ultra-pure H_2_O_2_ in Teflon Savillex^®^ beakers at 110 °C for 18h. Subsequently, the sample digests were evaporated to dryness at 90 °C and redissolved in 5 mL of (8 M *Optima* grade HCl + 0.001% H_2_O_2_). Chemical purification of the samples was achieved *via* a modification of the separation protocol^37^,^38^ using AG-MP1 anion exchange resin. For this, Bio-Rad Poly-Prep^®^ columns were filled with 1 mL of AG-MP1 resin. The resin was cleaned with 2 mL of 7 M HNO_3_ and 5 mL of 0.7 M HNO_3_, each time followed by rinsing with ~5 mL of Milli-Q H_2_O and it was subsequently conditioned with 5 mL of (8 M HCl + 0.001% H_2_O_2_). After sample loading, the matrix was removed using 3 mL of (8 M HCl + 0.001% H_2_O_2_), followed by the elution of Cu using 9 mL of (5 M HCl + 0.001% H_2_O_2_) and the removal of Fe and Zn using 0.6 M HCl and 0.7 M HNO_3,_ respectively. The Cu-fraction collected was evaporated to dryness at 90 °C, redissolved in 5 mL of (8 M HCl + 0.001% H_2_O_2_) and subjected to a second isolation on the same column using the same procedure. After this second isolation, the Cu-fraction was evaporated to dryness at 90 °C and redissolved in 1 mL of 14 M HNO_3_. This step was performed twice. The final residue was redissolved in 500 μL of 0.42 M HNO_3_.

In each batch of samples, blanks, a 1000 μg L^−1^ Cu in-house standard solution (Inorganic Ventures, lot C2-Cu02116) and the Seronorm^TM^ Trace Element Sero L-1 (SERO AS, lot 0903106) were included and treated in the same way.

The complete sample preparation procedure, including digestion, chemical purification and dilutions, was carried out in a class 10 clean room.

### Instrumentation and measurement protocols

Cu isotope ratio measurements were accomplished using a Neptune MC-ICP-MS instrument (Thermo Scientific, Germany), equipped with the Jet interface (X-type Ni skimmer and Jet type Ni sampler) for enhancing the sensitivity. The samples were introduced using a 100 μL min^−1^ PFA nebulizer and a dual spray chamber, consisting of a cyclonic and a Scott-type sub-unit. Measurements were performed using five Faraday collectors (connected to 10^11^ Ω amplifiers) in static mode. The instrument settings and data acquisition parameters are included in Supplementary Table S3. All measurements were performed at a mass position located 0.037 amu left from the center of the spectral peak plateau to avoid any effect of spectral interference present in the matrix.

Cu concentrations in the sample and isotopic standard solutions were adjusted to 200 μg L^−1^ and for some samples with low Cu levels to 100 μg L^−1^ Cu. For mass bias correction, the solutions were doped with Ni as internal standard. All samples and standards were measured following a sample-standard bracketing sequence. An in-house isotopic Cu standard previously characterized isotopically was included every 5 samples for checking the quality of the measurements.

Cu isotope ratios were determined off-line after 2 s.d. rejection of outliers. Correction for mass discrimination was performed using a combination of external correction in a sample-standard bracketing (SSB) approach and internal correction (with Ni) by means of the revised Russell’s law as described by Baxter *et al*.^39^. The isotopic composition of Cu is expressed in delta notation (per mil, ‰), calculated against the NIST SRM 976 Cu isotopic reference material using equation (1):


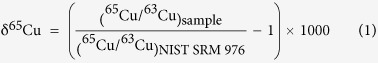


Elemental analysis was carried out using an Element XR sector field ICP-MS (SF-ICP-MS) instrument (Thermo Scientific, Germany) equipped with Ni cones (1.1 and 0.8 mm aperture diameter for the sampler and skimmer, respectively). Sample introduction was accomplished *via* a 200 μL min^−1^ quartz concentric nebulizer and a cyclonic spray chamber. The instrumental settings and data acquisition parameters used are also summarized in Supplementary Table S3. Levels of Cu and some elements that can give rise to spectral interference (Na and Mg) were determined both after digestion and after the isolation procedure. For quantitative elemental determination, external calibration was relied on with Ga as an internal standard.

### Method Validation

Purification of biological samples is highly recommended to avoid matrix-induced mass bias and spectral interference^40^. For Cu isotopic analysis in serum, the major problem arises from the presence of high levels of Na and, to a lesser extent Mg, leading to the occurrence of ^40^Ar^23^Na^+^ and ^40^Ar^25^Mg^+^, which can interfere with the monitoring of ^63^Cu^+^ and ^65^Cu^+^, respectively. For solutions containing 200 μg L^−1^ of Cu of the in-house standard, 200 μg L^−1^ of Ni and 1000 μg L^−1^ of Na, the average δ^65^Cu value, after mass bias correction according to Baxter’s method^39^, was 0.17 ± 0.03 (2 s.d.)‰ (n = 5). This value was in good agreement with the reference δ^65^Cu value for the in-house standard, i.e. 0.22 ± 0.07 (2 s.d.)‰^38^. After 2 subsequent isolations, the Na/Cu and Mg/Cu ratios (determined *via* SF-ICP-MS) observed in the Cu-fractions were always less than 1.5 and 1, respectively. Thus, no influence was observed on the Cu isotope ratio results under the conditions described.

Since chromatographic purification can induce on-column fractionation^41^, quantitative recovery is mandatory. For this reason, Cu levels were determined *via* SF-ICP-MS both after digestion and after isolation, yielding a Cu recovery of 95 ± 5% for all samples. The δ^65^Cu ± 2 s.d. (‰) values for the Seronorm^TM^ Trace Elements Serum L-1 reference material and the in-house standard were −0.24 ± 0.14 and 0.12 ± 0.13, respectively, both obtained after 4 different isolation and measurement sessions. The delta-value for the Cu in-house standard, measured every 5 samples, was always in good agreement with the expected value.

The procedural blanks were always measured at the beginning of each measurement session. As the bias between the results with and without blank correction was always less than 0.05‰, no blank correction was performed.

### Statistical Analysis

All statistical tests were performed using the IBM® SPSS Statistics 23 software for Windows. The related samples Wilcoxon signed rank test – *i.e.* a non-parametric alternative for the paired sample t-test – was performed for establishing differences in Cu levels and Cu isotope ratios in serum of patients pre- and post-LTx. The threshold for significance was defined as α = 0.05.

### Ethics Statement

This research project was approved by an independent commission for medical ethics connected to the Ghent University Hospital and was performed in accordance with the guidelines for good clinical practice and the statement of Helsinki, emplaced to protect volunteers participating in experiments. All patients signed an informed consent form concerning this study.

## Additional Information

**How to cite this article**: Lauwens, S. *et al*. Cu isotopic signature in blood serum of liver transplant patients: a follow-up study. *Sci. Rep.* **6**, 30683; doi: 10.1038/srep30683 (2016).

## Supplementary Material

Supplementary Information

## Figures and Tables

**Table 1:** Description of the patients’ etiology.

Disease	Number of patients
Ethylic C	11 (2 of which with HCC)
HCV induced C	5 (3 of which with HCC)
PSC	5
ISC	1
C (not specified)	2 (1 of which with HCC)
NASH	2 (2 of which with HCC)
HBV	2 (1 of which with HCC and 1 with HCC and PBC)
ALF	1
Polycystic liver	1
Familial amyloidosis	1
Non-cirrhotic portal hypertension	1
Total ESLD cohort	32 (10 of which with HCC)

C = cirrhosis, HCV = hepatitis C virus, PSC = primary sclerosing cholangitis, ISC = incomplete septal cirrhosis, NASH = non-alcoholic steato-hepatitis, HBV = hepatitis B virus, PBC = primary biliary cirrhosis.

**Table 2:** Liver function parameters (bilirubin, Alb, AST, ALT, PT and INR) pre-LTx and 3 months post-LTx.

Liver	Pre-LTx			3 months post-LTx			p-value	Normal range
parameter	Median	s.d.	Range (min–max)	Median	s.d.	Range (min–max)		
Bilirubin (mg dL^−1^)	3.0	9.8	0.3–46.6	0.60	0.86	0.2–4.1	0.000	0.2–1.1
Alb (g L^−1^)	34.0	7.8	22–49	37.0	8.4	19–44	> 0.05	34–48
AST (U L^−1^)	48	36	19–153	25	35	8–169	0.001	0–37
ALT (U L^−1^)	31	39	8–182	23	56	6–283	> 0.05	7–40
PT (%)	60	25	21–117	88	14	54–114	0.000	70–120
INR	1.43	0.72	0.9–3.7	1.10	0.12	0.9–1.5	0.000	0.9–1.1

The median, standard deviation and range (minimum value and maximum value observed) are represented for each cohort (pre-LTx and post-LTx). A significant change, i.e. normalization, in bilirubin, AST, PT and INR was observed 3 months post-LTX (p < 0.01, related samples Wilcoxon signed rank test). For clarity, also the normal ranges of these liver function parameters are shown.
