# Supplementary material for: Cu isotopic signature in blood serum of liver transplant patients: a follow-up study
Source: Sci Rep. 2016 Jul 29;6:30683. doi: 10.1038/srep30683 (PMC4965812; doi:10.1038/srep30683)
Supplement: Supplementary Information [file srep30683-s1.pdf]

Cu isotopic signature in blood serum of liver transplant patients: a follow-up study

Sara Lauwens, Marta Costas-Rodríguez, Hans Van Vlierberghe and Frank Vanhaecke

Table S1a: Liver function parameters and motivation for liver transplantation (LTx) for all patients. Bili = bilirubin, Alb = albumin, PT = prothrombin time, INR = international normalized ratio, AST = aspartate aminotransferase, ALT = alanine aminotransferase. – indicates that the data are not available. PSC = Primary sclerosing cholangitis. HCC = hepatocellular cancer, HBV = Hepatitis B Virus, HCV = Hepatitis C Virus, NASH = non-alcoholic steato-hepatitis, PBC = primary biliary cirrhosis, ADPKD = autosomal dominant polycystic kidney disease.

Patient	Date LTX D/M/Y	Sampling date D/M/Y	Date of birth D/M/Y	Bili (mg/dL)	Alb (g/L)	PT (%)	INR	AST (U/L)	ALT (U/L)	Motivation LTx
1	22/07/2014	22/07/2014	15/05/1956	5.70	25	42	1.93	32	27	PSC
		31/10/2014		-	-	69	1.25	11	10	
2	24/09/2011	24/09/2011	28/07/1951	4.40	26	50	1.70	113	52	Ethylic liver cirrhosis and HCC
		27/12/2011		0.60	43	75	1.2	103	178	
		11/06/2012		0.70	50	85	1.08	23	15	
		15/09/2014		0.80	47	87	1.09	27	18	
3	21/11/2011	10/11/2011	22/09/1962	0.70	37	66	1.38	39	21	HCV with cirrhosis and hepatopulmonal syndrome
		14/02/2012		0.20	43	71	1.30	26	28	
4	23/12/2011	30/11/2011	20/07/1969	7.30	23	32	2.59	91	59	Ethylic liver cirrhosis
		26/03/2012		0.20	-	114	0.96	26	31	
5	23/12/2012	23/12/2012	25/11/1961	2.30	34	70	1.23	41	27	Second LTx in 2012 due to non-cirrhotic portal hypertension
		22/01/2013		0.60	-	110	0.91	7	16	(Patient underwent first LTx in 2002 due to PSC)
		25/02/2013		0.50	43	105	0.94	8	14	
		23/03/2013		0.50	-	95	1.02	8	18	
		17/05/2013		0.60	-	93	1.03	10	17	
		28/06/2013		1.10	46	86	1.08	17	24	
		9/08/2013		1.10	-	103	0.97	14	11	
		4/09/2013		0.80	45	89	1.06	15	14	
		28/10/2013		1.00	45	99	0.99	17	23	
		17/02/2014		0.70	46	92	1.08	23	27	
6	23/05/2014	23/05/2014	26/01/1947	1.80	35	86	1.12	85	46	Ethylic liver cirrhosis and HCC
		25/08/2014		-	-	-	-	25	26	
7	2/01/2014	2/01/2014	23/05/1960	2.10	29	53	1.58	49	31	HCV liver cirrhosis and HCC
		4/04/2014		0.70	-	91	1.09	47	77	
		30/05/2014		2.50	-	78	1.19	28	44	
8	15/05/2013	13/05/2013	21/03/1949	3.00	48	60	1.4	81	66	NASH cirrhosis with HCC
		24/07/2013		0.50	30	79	1.14	40	16	
9	9/08/2013	9/08/2013	28/09/1947	3.20	33	45	1.76	19	8	Ethylic cirrhosis
		14/11/2013		1.70	32	79	1.14	14	7	
		25/02/2014		1.60	42	40	2.01	14	11	
		20/05/2014		1.80	42	90	1.09	12	9	
10	20/02/2014 AND 1/04/2015	19/02/2014	29/10/1957	2.70	31	81	1.17	71	51	PSC
		22/05/2014		1.30	32	88	1.1	35	23	

Table S1a: Continuation

11	4/03/2015	4/03/2015	6/05/1959	7.20	22	31	2.56	35	23	Ethylic cirrhosis
		5/05/2015		0.20	19	102	0.99	20	15	
12	29/10/2011	29/10/2011	19/05/1951	1.70	29	61	1.4	31	24	NASH liver cirrhosis with 2 HCC injuries
		7/02/2012		0.20	-	96	1.02	9	12	
		10/05/2012		0.20	42	94	1.04	9	10	
		5/07/2012		0.30	-	-	-	10	11	
13	13/11/2013	10/11/2013	5/09/1964	20.00	29	48	1.67	153	97	Ethylic cirrhosis
		7/02/2014		0.40	-	76	1.23	-	-	
14	30/11/2013	30/11/2013	3/07/1986	4.20	43	76	1.17	47	13	Incomplete septal cirrhosis with hepatopulmonal syndrome
		25/02/2014		0.50	-	100	1.02	27	26	
15	25/08/2014	24/08/2014	19/03/1962	-	41	104	0.94	58	52	PSC and auto-immune cholangitis
		22/11/2014		1.30	20	54	1.49	41	21	
16	10/07/2013	4/07/2013	10/10/1943	9.30	-	21	3.66	52	55	Liver cirrhosis and HCC
		18/09/2013		4.10	24	73	1.21	76	28	
17	27/11/2013	27/11/2013	1/12/1943	4.60	36	46	1.74	67	45	Ethylic cirrhosis
		25/02/2014		0.50	-	74	1.23	10	6	
		27/05/2014		0.80	43	65	1.36	12	11	
		4/09/2014		0.70	45	81	1.13	11	8	
18	6/01/2014	4/01/2014	12/11/1971	3.40	-	59	1.45	39	24	Liver cirrhosis (probably due to bariatric surgery)
		2/04/2014		1.20	-	-	-	27	22	
19	1/08/2014	31/07/2014	20/12/1943	0.40	44	90	1.03	30	22	HCV liver cirrhosis and HCC
		4/11/2014		0.20	44	94	1.03	22	23	
		13/05/2015		0.60	48	102	1.01	32	31	
20	13/12/2013	11/12/2013	6/04/1964	3.50	29	40	1.96	70	32	Ethylic cirrhosis
		1/04/2014		2.60	-	88	1.1	60	57	
		10/06/2014		1.50	43	82	1.09	30	30	
		3/09/2014		2.20	40	87	1.09	36	58	
21	11/01/2012	6/01/2012	21/12/1977	2.90	34	36	2.28	26	14	Ethylic liver cirrhosis and hepatorenal syndrome
		3/07/2012		1.00	34	69	1.24	102	66	
22	15/04/2013	15/04/2013	4/05/1952	0.60	22	61	1.39	28	18	Ethylic cirrhosis and portal hypertension
		23/07/2013		0.60	44	80	1.13	13	8	
		17/09/2013		0.50	-	86	1.08	16	12	
		28/01/2014		0.80	-	-	-	14	11	
23	14/07/2012	13/07/2012	22/07/1971	0.30	49	87	1.06	25	15	Polycystic liver due to ADPKD
		16/10/2012		0.40	42	107	0.93	36	64	
		15/04/2013		0.10	44	106	0.95	41	27	
		30/07/2013		0.20	-	98	1	41	18	
24	24/12/2013	24/12/2013	25/03/1965	1.60	29	53	1.55	72	34	HBV cirrhosis, PBC and HCC
		1/04/2014		-	-	-	-	18	12	
		17/06/2014		0.50	-	77	1.17	33	53	
		10/09/2014		0.40	47	77	1.15	17	15	

Table S1a: Continuation

25	24/11/2013	22/11/2013	9/03/1957	25.10	36	68	1.28	89	54	Ethylic cirrhosis
		24/02/2014		-	29	83	1.15	22	18	
		8/08/2014		0.70	-	-	-	30	34	
26	19/02/2013	18/02/2013	26/02/1970	0.60	37	117	0.89	39	30	Recidive PSC
		2/05/2013		0.60	40	98	1.00	18	19	
		26/11/2013		0.60	42	106	0.95	17	18	
27	15/01/2014	15/01/2014	24/06/1972	0.70	45	91	1.09	74	149	PSC with cirrhosis
		25/04/2014		0.70	-	114	0.95	24	37	
		20/01/2015		0.30	41	103	0.94	26	44	
28	11/07/2014	10/07/2014	31/07/1956	46.60	30	24	3.25	150	182	Acute liver failure and secundary acute renal failure
		14/10/2014		2.00	43	90	1.06	15	28	
		27/11/2014		1.80	46	89	1.04	12	28	
29	30/06/2014 and 13/07/2014	27/06/2014	13/05/1951	7.80	30	50	1.65	35	15	HCV liver cirrhosis
		14/10/2014		0.70	37	78	1.16	14	7	
		14/03/2015		-	-	63	1.36	-	-	
		27/07/2015		0.70	-	87	1.11	11	<5	
30	22/11/2013 and 25/11/2013	21/11/2013	9/05/1945	1.50	46	88	1.07	26	31	HBV, HCC resection on 11/01/2013
		25/02/2014		0.40	40	95	1.05	22	38	
		21/08/2014		0.50	45	78	1.13	44	90	
31	15/06/2013	15/06/2013	26/01/1948	22.70	35	23	3.35	121	102	HCV cirrhosis and HCC
		18/09/2013		1.00	-	88	1.07	66	39	
		11/03/2014		0.80	37	104	1.00	89	110	
		13/06/2014		-	-	-	-	86	101	
32	4/01/2014	2/01/1014	27/07/1972	0.40	45	98	1.00	26	22	Familial amyloidosis
		3/04/2014		0.40	-	84	1.13	169	283	
		6/10/2014		0.70	40	82	1.12	35	26	
		22/01/2015		0.30	44	97	0.98	35	22	
	Normal range			0.2-1.1	34-48	70-120	0.9-1.1	0-37	7-40	

Table S1b: Complications after LTx and other diseases

Patient	Complications after LTx	Other diseases
1	Klebsiella pneumonia. 09/2014: Deep vein thrombosis<br>09/2014: acute coronary syndrome	-
2	-	-
3	08/2012: Intracerebral hemorrhage	Psoriasis, diabetes mellitus<br>psoriasis-artrithis
4	06/2012: sclerosing cholangitis with periportal fibrosis of liver, hepatic artery aneurysm	-
5	02/2013: inguinal hernia repair<br>03/2013: acute renal failure<br>05/2013: acute to chronic renal failure<br>08/2013: Recidive acute renal failure	adrenocortical deficiency<br>1991: crohn's disease<br>1995: PSC<br>2003: total colectomy
6	06/06/2014: epileptic insult. 25/06/2014: Abnormal liver function parameters.<br>09/07/2014: acute renal failure, aortic valve replacement. 17/06/2015: inguinal hernia repair	diabetes mellitus type 2<br>bowen's disease
7	20/02/2014: Umbilical hernia, recidive HCV<br>28/04/2014: Start HCV trial, 13/10/2014: Stop HCV trial. HCV-RNA is negative on end of treatment	-
8	Ascites, portal hypertension, acute renal failure, neutro- and lymphopenia. Deceased on 25/07/2013	Diabetes mellitus
9	23/04/2014: incisional hernia repair<br>07/2014: Slow artrial fibrillation	-
10	01/03/2014: psychosis, 30/04/2014: abdominal pain, 13/10/2014: recidive cholangitis<br>01/04/2015: retranplantation	Colitis Ulcerosa
11	Good liver function and decrease in levels of transaminases.<br>Acute on chronic renal failure. Donor liver HBV positive. Sub-hepatic hemotoma.	-
12	25/01/2012: incisional hernia repair<br>07/2013: two vessel coronary artery disease<br>10/2013: Myelodysplastic syndrome (RCMD)<br>12/2012: omental infarction, 08/2014: Lupus anticoagulans	diabetes mellitus type 2
13	18/11/2013: perforation of the caecum, 02/12/2013: decubitus wound tail bone<br>Prolonged post-operative status: critical illness neuropathy with tetraparesis, atrial fibrillation, clostridium colitis, infectious orchitis, CMV reactivation.	-
14	02/12/2014: biliary leaks	Consanguineous parents
15	complicated postoperative status. 04/09/2014: biliary leaks, 23/10/2014: perforated bile duct anastomosis, 24/03/2015: hypovolemic shock, diarrhea, acute renal failure and hyperchloremic metabolic acidosis, bacteremia, acute renal failure, leukopenia, trombocytopenia, respiratory failure, multiple liver abscesses	Crohn's disease
16	Deceased on 18/09/2013 due to sepsis and hepatorenal syndrome	-
17	-	gonarthrosis
18	11/2014: Recidive liver failure with abnormal liver function parameters.<br>28/11/2015: Deceased	-
19	-	1956: epilepsy<br>12/2012: esophageal varices bleeding<br>03/2015: Herpes Zoster
20	Incisional hernia, 04/2014: stenosis anastomosis	-
21	05/2012: CMV reactivation	Epilepsy, congenital heart valve
22	10/2013: Echography shows symptoms of a steatotic liver. In general a good hepatic status.	2008: macrocytic anemia, vitamin B12<br>2012: chronic macrocytic anemia

Table S1b: Continuation

23	08/2012: gastroenteritis and abnormal liver function parameters.<br>12/2012: CMV reactivation, 02/2012: cholangitis.<br>31/05/2013: incisional hernia repair, recidive ascites, acute renal failure. 26/09/2013: ascites<br>05/2014: stricture on ductus hepaticus communis	-
24	08/04/2014: biliary leaks, 04/2015: acute appendicitis, 05/2015: fecal impaction	-
25	Respiratory failure due to restrictive lung disease and unilateral diaphragm paralysis leading to weaning problems<br>Clostridium difficile enterocolitis. Critical illness polyneuropathy.<br>Tracheostomy	-
26	28/4/2014: total colectomy for colitis ulserosa	Colitis Ulcerosa
27	-	1992: auto-immune hepatitis
28	-	Benign prostatic hypertrophy
29	13/07/2014: Retransplantation due to primary non function<br>Very prolonged operative status and pneumonia<br>03/2015: cytopenia, infection and , infectie, cerebrovascular accident (completely recovered)	-
30	2014: epilepsy, 06/2014: Pemphigus paraneoplastic<br>10/10/2014: hyperkalemia<br>11/2014: right hepatic duct stenosis	Arthrosis
31	16/06/2013: Hemoperitoneum, hemodialysis, polyneuropathy, pneumonia, narrowing right coronary artery<br>27/02/2014: cholangiopathy<br>09/2014: Lung-metastasized recidive HCC<br>12/12/2014: Deceased	Chronic pancreatitis<br>diabetes mellitus
32	Abnormal liver function tests<br>10/2014: cholangitis, acute on chronic renal failure	-

Table S2: Serum Cu concentration and Cu isotopic composition for each patient and sampling date.

Patient	Gender M/F	LTX Code	Date LTX D/M/Y	Sampling date D/M/Y	Cu (µg/L)	δ^{65}Cu(‰)	±	2s
1	M	ltx150	22/07/2014	22/07/2014	1258	-1.45	±	0.04
				31/10/2014	959	-0.44	±	0.11
2	M	ltx24	24/09/2011	24/09/2011	797	-1.42	±	0.02
				27/12/2011	1162	-1.54	±	0.01
				11/06/2012	881	-1.26	±	0.12
				15/09/2014	973	-1.73	±	0.03
3	M	ltx39	21/11/2011	10/11/2011	897	-1.31	±	0.02
				14/02/2012	483	-0.73	±	0.02
4	F	ltx32	23/12/2011	30/11/2011	273	-1.18	±	0.02
				26/03/2012	665	-0.51	±	0.05
5	M	ltx91	23/12/2012	23/12/2012	1043	-1.12	±	0.10
				22/01/2013	971	-0.95	±	0.05
				25/02/2013	985	-0.87	±	0.06
				23/03/2013	1194	-0.97	±	0.04
				17/05/2013	1106	-0.95	±	0.16
				28/06/2013	1016	-0.86	±	0.07
				9/08/2013	836	-0.76	±	0.13
				4/09/2013	925	-0.51	±	0.21
				28/10/2013	1064	-0.84	±	0.08
				17/02/2014	894	-0.59	±	0.09
6	M	ltx146	23/05/2014	23/05/2014	153	-1.11	±	0.04
				25/08/2014	953	-0.38	±	0.11
7	M	ltx133	2/01/2014	2/01/2014	793	-1.06	±	0.05
				4/04/2014	998	-1.08	±	0.16
				30/05/2014	823	-0.77	±	0.06
8	M	ltx104	15/05/2013	13/05/2013	792	-1.00	±	0.12
				24/07/2013	612	-0.55	±	0.02
9	M	ltx111	9/08/2013	9/08/2013	385	-0.95	±	0.02
				14/11/2013	882	-1.00	±	0.02
				25/02/2014	719	-0.70	±	0.01
				20/05/2014	771	-0.64	±	0.07
10	M	ltx142	20/02/2014 and 1/04/2015	19/02/2014	1739	-0.87	±	0.03
				22/05/2014	920	-0.86	±	0.01
11	M	ltx165	4/03/2015	4/03/2015	449	-0.86	±	0.02
				5/05/2015	386	-0.51	±	0.03
12	M	ltx36	29/10/2011	29/10/2011	474	-0.85	±	0.02
				7/02/2012	1079	-0.59	±	0.04
				10/05/2012	927	-0.68	±	0.02
				5/07/2012	677	-0.63	±	0.08
13	M	ltx122	13/11/2013	10/11/2013	501	-0.83	±	0.02
				7/02/2014	575	-0.84	±	0.02
14	M	ltx128	30/11/2013	30/11/2013	846	-0.81	±	0.07
				25/02/2014	978	-0.82	±	0.05
15	F	ltx153	25/08/2014	24/08/2014	1055	-0.76	±	0.05
				22/11/2014	262	-0.30	±	0.02
16	M	ltx110	10/07/2013	4/07/2013	152	-0.74	±	0.03
				18/09/2013	479	-0.93	±	0.13
17	M	ltx127	27/11/2013	27/11/2013	774	-0.74	±	0.00
				25/02/2014	762	-0.63	±	0.02
				27/05/2014	655	-0.26	±	0.02
				4/09/2014	759	-0.29	±	0.02
18	F	ltx137	6/01/2014	4/01/2014	79	-0.72	±	0.05
				2/04/2014	196	-0.40	±	0.17

Table S2: Continuation

19	M	ltx152	1/08/2014	31/07/2014	1146	-0.71	±	0.09
				4/11/2014	916	-0.55	±	0.02
				13/05/2015	1238	-0.43	±	0.11
20	M	ltx129	13/12/2013	11/12/2013	622	-0.68	±	0.02
				1/04/2014	846	-0.64	±	0.01
				10/06/2014	596	-0.36	±	0.09
				3/09/2014	936	-0.35	±	0.02
21	F	ltx42	11/01/2012	6/01/2012	390	-0.67	±	0.02
				3/07/2012	372	-0.50	±	0.03
22	M	ltx103	15/04/2013	15/04/2013	300	-0.63	±	0.02
				23/07/2013	938	-0.51	±	0.05
				17/09/2013	912	-0.36	±	0.02
				28/01/2014	798	-0.16	±	0.04
23	F	ltx64	14/07/2012	13/07/2012	1218	-0.58	±	0.13
				16/10/2012	1098	-0.84	±	0.04
				15/04/2013	1153	-0.58	±	0.06
				30/07/2013	680	-0.27	±	0.15
24	M	ltx132	24/12/2013	24/12/2013	1067	-0.57	±	0.02
				1/04/2014	832	-0.62	±	0.02
				17/06/2014	820	-0.02	±	0.07
				10/09/2014	851	-0.07	±	0.18
25	M	ltx125	24/11/2013	22/11/2013	740	-0.54	±	0.08
				24/02/2014	1209	-0.43	±	0.10
				8/08/2014	880	-0.23	±	0.01
26	M	ltx98	19/02/2013	18/02/2013	1378	-0.52	±	0.02
				2/05/2013	1059	-0.44	±	0.08
				26/11/2013	1060	-0.29	±	0.03
27	M	ltx138	15/01/2014	15/01/2014	961	-0.52	±	0.08
				25/04/2014	731	-0.30	±	0.07
				20/01/2015	861	-0.37	±	0.03
28	M	ltx149	11/07/2014	10/07/2014	687	-0.51	±	0.02
				14/10/2014	648	-0.18	±	0.09
				27/11/2014	919	-0.58	±	0.03
29	M	ltx147	30/06/2014 and 13/07/2014	27/06/2014	1066	-0.50	±	0.11
				14/10/2014	1078	-0.71	±	0.06
				14/03/2015	774	-0.78	±	0.03
				27/07/2015	952	-0.73	±	0.03
30	M	ltx124	22/11/2013 and 25/11/2013	21/11/2013	755	-0.49	±	0.02
				25/02/2014	904	-0.48	±	0.03
				21/08/2014	908	-0.35	±	0.01
31	M	ltx107	15/06/2013	15/06/2013	507	-0.49	±	0.02
				18/09/2013	548	-0.53	±	0.03
				11/03/2014	992	-0.53	±	0.07
				13/06/2014	864	-0.96	±	0.03
32	M	ltx113	4/01/2014	2/01/1014	683	-0.40	±	0.09
				3/04/2014	1333	-0.50	±	0.02
				6/10/2014	999	-0.75	±	0.14
				22/01/2015	821	-0.72	±	0.12

Table S3: Instrument settings and data acquisition parameters for the Neptune MC-ICP-MS and Element XR SF-ICP-MS instruments.

Neptune MC-ICP-MS	
Sample uptake rate (µL min^{-1})	100
Ar gas flow rates (L min^{-1})	Plasma 15, auxiliary 0.70, nebulizer 0.9-1.0
RF power (W)	1250
Guard electrode	Connected
Integration time (s)	4.194
Number of blocks	9
Number of cycles/block	5
Cup configuration	L3: ^{60}Ni; L1: ^{61}Ni; C: ^{62}Ni; H1: ^{63}Cu; H3: ^{65}Cu
Element XR SF-ICP-MS	
Sample uptake rate (µL min^{-1})	200
Ar glas flow rates (L min^{-1})	Plasma 15, auxiliary 0.95, nebulizer 1.0-1.1
RF power (W)	1200
Guard electrode	Connected
Acquisition mode	E-scan
Dwell time per point (ms)	10
Points per peak	20
Number of runs and passes	5 x 5
Nuclides monitored	^{63}Cu, ^{65}Cu, ^{64}Zn, ^{66}Zn, ^{24}Mg, ^{23}Na, ^{69}Ga
